# PI3K/AKT/mTOR Dysregulation and Reprogramming Metabolic Pathways in Renal Cancer: Crosstalk with the VHL/HIF Axis

**DOI:** 10.3390/ijms24098391

**Published:** 2023-05-07

**Authors:** Silviu Constantin Badoiu, Maria Greabu, Daniela Miricescu, Iulia-Ioana Stanescu-Spinu, Radu Ilinca, Daniela Gabriela Balan, Andra-Elena Balcangiu-Stroescu, Doina-Andrada Mihai, Ileana Adela Vacaroiu, Constantin Stefani, Viorel Jinga

**Affiliations:** 1 Department of Anatomy and Embryology, Faculty of Medicine, Carol Davila University of Medicine and Pharmacy, 8 Eroii Sanitari Blvd, 050474 Bucharest, Romania; silviu.badoiu@umfcd.ro; 2 Department of Biochemistry, Faculty of Dentistry, Carol Davila University of Medicine and Pharmacy, 8 Eroii Sanitari Blvd, Sector 5, 050474 Bucharest, Romania; maria.greabu@umfcd.ro; 3 Department of Medical Informatics and Biostatistics, Faculty of Dentistry, Carol Davila University of Medicine and Pharmacy, 8 Eroii Sanitari Blvd, 050474 Bucharest, Romania; radu.ilinca@umfcd.ro; 4 Department of Physiology, Faculty of Dentistry, Carol Davila University of Medicine and Pharmacy, 8 Eroii Sanitari Blvd, 050474 Bucharest, Romania; daniela.balan@umfcd.ro (D.G.B.); andra.balcangiu@umfcd.ro (A.-E.B.-S.); 5 Department of Diabetes, Nutrition and Metabolic Diseases, Faculty of Medicine, Carol Davila University of Medicine and Pharmacy, 8 Eroii Sanitari Blvd, 050474 Bucharest, Romania; andrada.mihai@umfcd.ro; 6 Department of Nephrology, Faculty of Medicine, Carol Davila University of Medicine and Pharmacy, 020021 Bucharest, Romania; ileana.vacaroiu@umfcd.ro; 7 Department of Family Medicine and Clinical Base, Dr. Carol Davila Central Military Emergency University Hospital, 134 Calea Plevnei, 010825 Bucharest, Romania; constantin.stefani@umfcd.ro; 8 Department of Urology, “Prof. Dr. Theodor Burghele” Hospital, 050653 Bucharest, Romania; 9 “Prof. Dr. Theodor Burghele” Clinical Hospital, University of Medicine and Pharmacy Carol Davila, 050474 Bucharest, Romania; 10 Medical Sciences Section, Academy of Romanian Scientists, 050085 Bucharest, Romania

**Keywords:** PI3K/AKT/mTOR, renal cancer, metabolism, VHL/HIF axis

## Abstract

Renal cell carcinoma (RCC) represents 85–95% of kidney cancers and is the most frequent type of renal cancer in adult patients. It accounts for 3% of all cancer cases and is in 7th place among the most frequent histological types of cancer. Clear cell renal cell carcinoma (ccRCC), accounts for 75% of RCCs and has the most kidney cancer-related deaths. One-third of the patients with ccRCC develop metastases. Renal cancer presents cellular alterations in sugars, lipids, amino acids, and nucleic acid metabolism. RCC is characterized by several metabolic dysregulations including oxygen sensing (VHL/HIF pathway), glucose transporters (GLUT 1 and GLUT 4) energy sensing, and energy nutrient sensing cascade. Metabolic reprogramming represents an important characteristic of the cancer cells to survive in nutrient and oxygen-deprived environments, to proliferate and metastasize in different body sites. The phosphoinositide 3-kinase-AKT-mammalian target of the rapamycin (PI3K/AKT/mTOR) signaling pathway is usually dysregulated in various cancer types including renal cancer. This molecular pathway is frequently correlated with tumor growth and survival. The main aim of this review is to present renal cancer types, dysregulation of PI3K/AKT/mTOR signaling pathway members, crosstalk with VHL/HIF axis, and carbohydrates, lipids, and amino acid alterations.

## 1. Renal Cancer Types and Renal Cancer Incidence

Renal cancer is a group of several tumors that develop in the kidney, each with a unique histology and clinical evolution, each responding differently to treatment, and each determined by a different gene mutation. Recently, the crucial role that metabolic pathways play in cancer has been emphasized. Moreover, research shows that the genes whose mutations are associated with renal cancer have interactions with the cell metabolism pathways for energy, nutrition, iron, or oxygen sensing [1].

Classically, any tumor is described on a histological basis. The World Health Organization postulates the shift of the diagnostic/classification criteria of renal tumors from exclusive morphologic criteria towards combined (integrated) criteria: clinical, imagistic, histologic, immunohistochemistry, and molecular (molecular markers, proteomics, study of the tumor microenvironment) [2,3,4].

Morphological criteria, which include the anatomical location (collecting duct carcinomas, juxtaglomerular cell tumors, renal medullary carcinomas) and the histology by cytoplasmic coloration (clear cell renal carcinomas, eosinophilic renal carcinomas, chromophobe renal carcinomas), cell form (spindle cell carcinoma), histological type of tissue involved in the tumoral process (epithelial renal cancers, stromal renal cancers, mesenchymal renal cancers) can be used to define and classify renal tumors [5]. 

Molecular and genetic criteria such as loss of alleles of loci on the short arm of chromosome 3 in renal cell carcinomas [6,7], genetic abnormalities in renal cell carcinomas, involving chromosomes 7 and 17 [8,9,10,11], multiple chromosomal losses and/or abnormalities in chromophobe renal cell carcinomas [12,13] are also included to define and classify renal malignancies. 

Additionally, renal cancer can be described based on the existence of correlations with specific renal diseases (acquired cystic disease–associated renal carcinomas) or with syndromes that have familial aggregation (hereditary leiomyomatosis and renal cell carcinoma syndrome–associated renal cell carcinoma) [14,15]. Table 1 presents the latest combined IACR/IARC/WHO classification of renal tumors [5].

### 1.1. Renal Cell Carcinoma (RCC)

RCC develops from the renal tubular epithelium or the renal cortex [16]. It represents 85–95% of kidney cancers, being the most frequent type of renal cancer in adult patients [17]. It accounts for 3% of all cancer cases and is in 7th place among the most frequent histological types of cancer [18] and worldwide affects over 400,000 patients every year [19]. North America and the Czech Republic are reported to have the highest incidence rates [20]. RCC was discovered at any age, but the sixth decade of life seems to be the most affected [20]. It concerns more frequently in men than women (2/1 ratio) [21] and includes more than 10 histological and molecular subtypes of renal cancers [22].

The major types of RCC are considered those with over 5% incidence each: clear cell renal cell carcinoma (ccRCC), papillary renal cell carcinoma (PRCC), and chromophobe renal cell carcinoma (ChRCC) [23,24,25,26]. The rare types of RCC account for less than 1% of incidence each [22]. There are, also, RCCs that do not comply with the histologic diagnostic criteria of the above-mentioned types (major types or rare types); these are called unclassified renal cell carcinomas (uRCCs) and they have all together less than 4% incidence [27,28].

#### 1.1.1. Clear Cell Renal Cell Carcinoma (ccRCC) 

ccRCC represents 75% of the RCCs and accounts for most kidney cancer-related deaths. Moreover, one-third of the patients with ccRCC develop metastases [24,29,30]. It involves patients in the 5th–7th decades of life [29,30]. Some of the ccRCCs associate with genetic disorders, the most prevalent being the *von Hyppel-Lindau* (*VHL*) gene (located on the short arm of chromosome 3) and the protein *polybromo-1* (*PBRM-1*) gene (also located on the short arm of the chromosome 3). Apparently, 95% of the ccRCCs exhibit a deletion in 3p (the short arm of chromosome 3) [31]. In addition to the genetic factors, there are acquired factors incriminated in the development of ccRCCs, such as smoking, long-term dialysis, obesity, diabetes mellitus, chronic administration of analgesic medication, and arterial hypertension [30].

ccRCCs develop from the proximal nephron and tubular epithelium [30] and appear as yellowish solid tumors with internal bleeding, necrosis, calcifications, and cystic degeneration. Because the cytoplasm contains large amounts of glycogen and lipids, the histological aspect is clear cells [32,33]. The morphological characteristics (histological grade, tumor size, tumor necrosis, tumoral grading, vascular invasion) are currently the most important prognostic factors of survival in ccRCCs [34].

#### 1.1.2. Papillary Renal Cell Carcinoma (PRCC)

PRCC accounts for 15% of all renal cancers, being the second most frequent kidney malignancy after ccRCC [35]. PRCC develops in the nephron, from the tubular epithelium, usually after the fifth decade of life. The prognosis of PRCC is similar to that of ccRCC [36].

Histologically, PRCC has papillae with vascular cores, foamy histiocytes, and psammoma bodies (round microscopical calcifications) [35]. Papillary tumors appear as long finger-like cell proliferations [35]. Classically, based on staining characteristics, PRCCs are divided into Type 1 (with basophilic cytoplasm) and Type 2 (with eosinophilic cytoplasm) [35,37]. 

Type 1 PRCC is characterized by gains of 7, 8q, 12q, 16p, 17, and 20 chromosomes and loss of 9p chromosome [11]. Frequently, the mutated gene is *MET* proto-oncogene (the MET tyrosine kinase domain is altered) [11], located on chromosome 7. Basophilic tumors are classified as low-grade [38]. 

Type 2 PRCC is characterized by gains of 8q chromosome, and loss of 1p and 9p chromosomes [11]. Frequently, the mutated genes are *CDKN2A* gene (cyclin-dependent kinase inhibitor 2A), *SETD2* gene (SET domain-containing 2)-the SET domain is a protein domain with methyltransferase activity, and *NRF2* gene (nuclear factor E2-related factor 2) [11]. Eosinophilic tumors are classified as high-grade [38].

Subclassification of PRCC into Type 1 and Type 2 appears to be no longer recommended and tumors previously classified as PRCC type 2 are presently considered individual entities [39].

#### 1.1.3. Chromophobe Renal Cell Carcinoma (ChRCC)

ChRCC represents about 5% of RCCs [40] and develops in the distal regions of the nephron. This type of RCC has a better prognosis than ccRCC [41], with its’ mortality being around 10%. It is diagnosed more frequently after the fifth decade of life [40].

Histologically, there is the classic type ChRCC (tumor cells exhibiting prominent membrane and pale cytoplasm) and the eosinophilic type ChRCC (large tumor cells with cytoplasm containing fine eosinophilic granules) [40]. Both types present similar cytogenetic alterations, such as the loss of chromosomes 1, 2, 6, 10, 13, 17, and 21, the mutated genes are *TP53* gene (Tumor Protein P53 gene) and *PTEN* gene (Phosphatase and TENsin homolog gene) [41].

#### 1.1.4. Clear Cell Papillary Renal Cell Carcinoma (ccpRCC)

ccpRCC is the 4th most prevalent type of RCCs and is diagnosed more frequently after the 7th decade of life. The clinical course of the tumor is considered to be indolent [42].

Histologically, it has specific features such as low-grade nuclei arranged horizontally and apically, distant from the basal membrane, but also morphological aspects similar to ccRCC and other morphological traits similar to PRCC [42]. It is agreed that specific nuclear arrangements associated with papillary architecture (focal or diffuse) are indicative of ccpRCC [43,44].

ccpRCC was renamed clear cell papillary renal cell tumor (ccPRCT) [39]. This switch from carcinoma toward tumor was performed because of the indolent behavior of this group of tumors [39,42]. The absence of recurrent cytogenetic abnormalities or the absence of *VHL* gene alterations is differentiation criteria from ccRCCs and PRCCs.

#### 1.1.5. Collecting Duct Carcinoma (CDC)

CDC are rare tumors, representing 2% of all renal malignancies [45]. This type of renal cancer develops between the 2nd–9th decades of life, being reported more frequently in men (men:women ratio is 2:1). It develops from the epithelium of the renal collecting ducts, and it is highly aggressive, having a mortality rate of 70% in two years [18]. Moreover, 71% of the patients with kidney-related clinical signs (the most typical being macroscopic hematuria) already have metastases [45].

Histologically, CDC is relatively polymorphic: tubulopapillary pattern/cords or tubular structures/columnar pattern/desmoplastic stroma/mucinous material [18,46]. CDC presents as cytogenetic alterations gains at chromosome 13q and losses of chromosomes 8p, 16p, 1p, and 9p [18,46]. However, generally, CDC is defined by a “lack of consistency and specificity of molecular profile” [46].

#### 1.1.6. Renal Medullary Carcinoma (RMC)

Renal medullary carcinoma is very rare, representing 0.5–1% of the RCCs [30,47] and it develops more frequently in the 2nd–3rd decades of life, with a higher incidence in Mediterranean and African males [48]. Additionally, it has been observed that patients with sickle cell disease are at increased risk to develop RMC [47,48,49]. This type of carcinoma develops from the distal nephron and is extremely aggressive, with the 3-year survival rate being less than 5% [47,50].

Histologically, RMC is very heterogenous, presenting many patterns—glandular, tubular, tubulopapillary, microcystic, adenoid cystic-like, and reticular—as poorly differentiated eosinophilic cells and inflammatory infiltrative cells [18,47,50].

The affected genes in RMC are *SMARCB1* (Hsieh, JJ 2017) and *INI1* [47]. The *SMARCB1* gene codifies the SMARCCB1 protein (related matrix-associated actin-dependent regulator of chromatin subfamily B member 1), which is a core subunit of the SWI/sucrose non-fermenting (SNF) ATP-dependent chromatin remodeling complex, that relieves repressive chromatin structures [18]. 

On the other hand, the *INI1* gene also loses its expression. *INI1* codifies the INI1 protein (Integrase Interactor 1), a protein capable of interacting with the integrase protein of the human immunodeficiency virus. SMARCB1/INI1 is one of the core subunit proteins of the ATP-dependent SWI/SNF chromatin remodeling complex [47] and it has an important tumor suppressor action [51,52].

#### 1.1.7. Unclassified Renal Cancers (uRCCs)

uRCCs represent 3–5% of the RCCs [30] and have a high mortality rate [18], affecting any age [30]. Histologically, uRCCs might share characteristics with other RCCs, or do not resemble any other type [53]. The genomic alterations encountered in uRCCs might be identified in other RCCs, but there are also rare subtypes with particular altered genes [54].

### 1.2. Urothelial Carcinoma (UC)

Urothelial carcinoma is a cancer that develops in the urothelium and accounts for almost 90% of all cases of bladder cancer and 7% of all cases of kidney cancer, including cancer of the renal pelvis and the ureter [55]. Urinary bladder urothelial carcinoma (UBUC) presents micropapillary sarcomatoid, squamous, and glandular variations. It is accessible to TUR (transurethral resection) and local chemotherapy; hence, it has a good prognosis [56,57,58].

### 1.3. Nephroblastoma or Wilms’ Tumor (WT)

Nephroblastoma is the most frequent kidney cancer in children and the most frequent abdominal cancer in children [59,60]. It is extremely rarely encountered in adults, the highest incidence being in the first decade of life (between 2 and 5 years of age) [61]. The highest incidence is in Africans and African Americans, while the lowest incidence is in East Asians (who also have a better prognosis). The incidence in North America and Europe is almost the same [61]. 

Wilms’ tumor may develop due to genetic alterations. Sometimes, the tumor is associated with specific groups of other signs and symptoms, resulting in a syndrome [30,60]. 

Frequently, the affected genes are *WT1* and *WT2*. *WT1* gene (Wilms’ Tumor 1 gene) is located on chromosome 11p13 and is essential for normal embryologic development of the kidney and genitourinary tract. This gene is deleted in WAGR syndrome (Wilms’ tumor, aniridia, genitourinary anomalies, intellectual disability) [62,63]. *WT1* gene is muted in Denys–Drash syndrome (Wilms’ tumor, gonadal dysgenesis, nephropathy) [62,63]. At least 50% of the Wilms’ tumors with affected *WT1* gene display acquired somatic mutations in *CTNNB1* gene (Catenin Beta 1 gene), which is located on chromosome 3 (3p22.1) [64].

*WT2* gene (Wilms’ Tumor 2 gene) is located on chromosome 11p15. The abnormal regulation of chromosome 11p15.5 is related to Beckwith–Widemann syndrome (macrosomia, macroglossia, hemihypertrophy, omphalocele, visceromegaly, Wilms’ tumor) [65,66]. We must note that most cases of Wilms’ tumor do not exhibit mutations of the above-mentioned genes [67].

Wilms’ tumor develops, in most situations, from metanephrogenic tissue, with metanephrogenic cells being found in 35% of unilateral and 100% of bilateral Wilms’ tumors [68]. The histological type is the most important prognostic factor in Wilms’ tumor [69]. Based on the cell types, there are two histological types of nephroblastoma, the classical type, with a favorable prognosis, and the anaplastic type, with an unfavorable prognosis. 

The classical type nephroblastoma accounts for 92–95% of Wilms’ tumors and, due to its favorable histology, has a good prognosis (with correct treatment) [69]. It has a triphasic histological pattern, including metanephrogenic blastema derivatives, epithelial derivatives, and stromal derivatives, in variate proportions [70,71]. Metanephrogenic blastema represents the less differentiated and most malignant component. 

The anaplastic type nephroblastoma accounts for 5–8% of Wilms’ tumors and, due to its unfavorable histology, has a poor prognosis [70,71]. The anaplasia is defined by nuclear enlargement, multiple mitoses, pleomorphism, and hyperchromatic nuclei [72,73]. The anaplasia can be focal (a localized area with anaplastic features, surrounded by non-anaplastic cells) or diffuse (all the other situations) [72,73]. 

In high-income countries, with adequate treatment, the 5-year survival rate in children with a classical Wilms’ tumor is 92% (Northern America). At the same time, in low-income countries, the 5-year survival rate drops to 78% [61]. The anaplastic Wilms’ tumor has a worse prognosis because of its increased chemoresistance [74,75]. 

### 1.4. Renal Sarcoma

Renal sarcoma is a rare kidney tumor, accounting for 1–2% of renal malignant tumors [76]. It develops (initially) in the connective tissue and renal blood vessels (renal capsule and perisinuous space). The origin of renal sarcomas is represented by the mesenchymal cells, which is why they expand easily and cross anatomical boundaries (capsulae, fasciae) [76]. 

Histological types are represented by leiomyosarcomas (LMSs) (50–60% of renal sarcomas), liposarcomas (10–15% of renal sarcomas), and rare variants such as Ewin’s sarcoma/primitive neuroectodermal tumor (PNET), interdigitating dendritic cell sarcoma (IDCS), malignant hemangiopericytoma, malignant fibrous histiocytoma, angiosarcoma, anaplastic sarcoma, myeloid sarcoma, osteogenic sarcoma, synovial sarcoma, rhabdomyosarcoma (RMS), fibrosarcoma, carcinosarcoma [77]. 

LMSs develop usually in the 4th–6th decades of life and affect women more frequently. The tumor is unilateral in the majority of reported cases, involving especially the right kidney [78]. The histological aspect of renal sarcomas might resemble other renal cancers, but, classically, there are trabeculae or nests made of plump ovoidal cells, separated by fibrovascular septa [77,78].

Genetically, renal sarcomas are considered to have “chaotic” karyotypes, presenting several genetic alterations. Smaller tumors are associated with the overexpression of *PRUNE 2* gene (*prune homolog 2 with BCH domain*) [79] and thus of having a better prognosis, while in large tumors *PRUNE 2* gene is downregulated, which is correlated with a worse prognosis. Alterations of *FH* (*Fumarate Hydrase*) gene [80,81] can also be present in renal sarcomas. At the same time, 17q duplication is associated with low-risk metastases, longer survival, and better prognosis [82]. Longer survival was also found to be correlated with 1p33-p32.3 duplications [83]. On the other hand, 1q21.3 duplications are associated with shorter survival [83], while 4q31 and 18q22 deletions are associated with an increased risk of metastases [84]. LMSs (pelvic and retroperitoneal, including kidneys) can display mutations of the *MED 12* gene (*mediator of RNA polymerase II transcription subunit 12* homolog gene) [85] or overexpression of p16 and p53 tumor suppressor proteins [86].

Overall, the prognosis of renal sarcomas is not easily predictable, because of multiple histological subtypes with variable genetic alterations. Moreover, epidemiological studies suggest that these types of tumors have high metastatic potential and poor prognosis [87].

## 2. PI3K/AKT/mTOR

### 2.1. Description of PI3K/AKT/mTOR Signaling Pathway

A wide range of cellular processes, including survival, proliferation, growth, metabolism, angiogenesis, and metastasis, are regulated by the phosphatidylinositol-3-kinase PI3K/AKT/mammalian target of rapamycin (mTOR) signaling pathway, which is overactivated in different cancer types by molecular abnormalities [88,89,90].

The basic biological activity of the PI3K family of lipid kinases is to phosphorylate the 3-hydroxyl group of phosphoinositides [91]. There are three classes (I-III) of PI3K, each with a distinct structure and preferred substrate [91,92]. Class I PI3Ks are heterodimers made up of a regulatory and a catalytic subunit and are represented by p85 and p110 (α, β, and δ) which are included in the class IA group and p101 and p110 γ in the class IB PI3K [93]. These four members of the class I PI3K are encoded by *PIK3CA*, *PIK3CB*, *PIK3CG*, and *PIK3CD* and facilitate the phosphorylation of PtdIns-4,5-P2 to produce PtdIns-3,4,5-P3 (PI(3,4,5)P3)), which will promote the recruitment of cytoplasmic proteins, acting as a second messenger [94]. 

Due to its frequent alterations, *PIK3CA* is most involved in human malignancies related to breast and colon cancer. *PIK3CB* gene mutation is rarer, although it is expressed ubiquitously. *PIK3CD* is mostly expressed in white blood cells and B cells and is crucial for the survival and maturation of B cell follicles, while *PIK3CG* expression is inversely correlated with colon cancer growth, despite the fact that PIK3CG levels are connected to cancer growth [95].

p110α, encoded by *PIK3CA* on chromosome 3 at 3q26.3, p110β by *PIK3CB* at 3q23, and p110δ are PI3-kinases that interact with a family of Src homology 2(SH2)-domain-containing regulatory adaptor proteins which facilitate their activation by growth factor receptors [96]. Studies show that p110α isoform can influence the size of the adult heart and has an important role in the survival of the cell, while p110β promotes proliferation [97]. 

*PIK3CG* at 7q21.11 encodes p110γ, a catalytic subunit that lacks a p85 binding site, which is the only PI3K class IB member [98] together with its adaptor protein, p101, encoded by PI3K regulatory subunit gene at 17p13.1 [99]. Gβγ subunits and also, possibly, Gα subunits promote activation of class IB, which plays a key role as a modulator of inflammation, in heart contractility and allergies [95,100]. 

Growth factor stimulation causes PtdIns-3,4,5-P3 to be rapidly produced in healthy cells. Lipid phosphatases, such as the tumor suppressor PTEN, quickly break down this compound and stop PI3K signaling by removing the 3′-phosphate [101,102]. Due to the enhanced activity of oncogenic signaling proteins located upstream of PI3K or due to mutational activation of PI3K itself, cancer cells commonly have elevated levels of PtdIns-3,4,5-P3. Many malignancies also show loss of *PTEN* function, which increases basal and stimulated PtdIns-3,4,5-P3 abundance by slowing down the second messenger’s turnover rate. Furthermore, *PIK3CA* and *PTEN* were discovered to be the second and third most altered genes in human tumors [101]. 

Upon receptor activation, PI(3,4,5)P_3_ and PI(3,4)P_2_ bind to a variety of effectors that have pleckstrin homology (PH) domains and collectively control an intricate signaling network, which can regulate the activity of tiny guanosine triphosphatases and protein kinases that govern cellular adhesion, movement, contraction, and secretion [103].

The core of class II PI3Ks (PI3K-C2α, PI3K-C2 β, and PI3K-C2γ) consists of a central C2 domain, a helical domain, and a bilobal kinase, just like class I and class III PI3Ks. These enzymes can generate both phosphatidylinositol 3-phosphate (PtdIns(3)P) and PtdIns(3,4)P2). One of the three class II PI3K isoforms, class II alpha PI3K (PI3K-C2α), encoded by *PI3KC2A* at 11p15.5-p14 [96], is expressed in mammals and is crucial for embryonic development. Moreover, PI3K-C2α is essential for clathrin-mediated endocytosis, insulin production and signaling, angiogenesis, and primary cilium function. At the same time, research using animal models linked to pathologies such as cancer, diabetes, vascular disease, and thrombosis with loss of function of this isoform of PI3K class II [104]. 

Recent studies using super-resolution imaging demonstrated that only PI3K-C2β was strongly colocalized to actin filament (F-actin)-associated clathrin-coated pits and vesicles, despite both PI3K-C2β and PI3K-C2α being co-localized to clathrin-coated pits and vesicles. Based on these findings, it appears that the two class II isoforms participate in clathrin-mediated pinocytosis differently [105]. Another study revealed that mitotic progression is regulated by PI3K-C2β, which is encoded by *PIK3C2B* at 1q32 [96,106]. PI3K-C2β levels that are higher than normal have been found in primary neuroblastoma tumors and cell lines. A group of variations in the promoter and upstream regions of the *PIK3C2B* gene, which codes for PI3K-C2β, have been linked to an increased risk of prostate cancer. Additionally, the involvement of PIK3-C2β has also been discovered in lung cancer, ovarian and cervical cancer [107,108]. Moreover, a recent study performed on human breast cancer cell lines and human breast cancer tissues revealed elevated levels of PI3K-C2β, showing that there is a correlation between this class II isoform and the tumor’s proliferative capacity. Additionally, PI3K-C2β downregulation prevented breast cancer metastasis development in vivo and inhibits the invasion of breast cancer cells in vitro [107]. 

Currently, there is very little information regarding the role of PI3KCAγ in malignancy. Nevertheless, there is research suggesting the involvement of this isoform encoded by *PIK3C2G* at 12p12 in ovarian cancer, pancreatic ductal adenocarcinoma, and leukemia [96,109]. 

Class III of PI3Ks has a sole representative, Vps34, an enzyme encoded by *PI3KC3* chromosome 18 at position 18q12.3. This PtdIns3-kinase is primarily found on intracellular membranes and was first discovered as a mutation of vesicle-mediated vacuolar protein sorting in yeast. PI3K class III promotes endosome fusion during intracellular trafficking events and is engaged in a variety of intracellular trafficking processes, such as autophagy and phagosome formation, retrograde endosome to Golgi transport, and transport at the nuclear membrane [110,111,112,113]. By attaching to a protein complex made up of a regulatory subunit and a catalytic subunit, PIK3C3 regulates autophagy and macrophage phagocytosis (Table 2) [114]. 

The PI3K pathway’s main effector is AKT (a serine/threonine kinase), which has a number of downstream effectors that control important cellular functions [115,116]. AKT, also known as protein kinase B (PKB), exists in mammals in three different isoforms: AKT1, AKT2, and AKT3. While AKT3 is largely expressed in the brain and has relatively limited expression due to its tissue distribution, AKT1 and AKT2 are abundant in numerous tissues, including pancreatic tissue [117].

These isoforms have distinct and important functions in cancer: AKT2 promotes cell migration and invasion, while AKT3 is linked to hormone independence. Furthermore, *AKT2* gene is overexpressed in pancreatic cancer, while breast and prostate cancers present *AKT3* gene overexpression [118,119].

By participating in several different signaling pathways in the human body, the mammalian Target of Rapamycin (mTOR) controls cell division, autophagy, and apoptosis, being linked to insulin resistance, osteoporosis, cancer, and rheumatoid arthritis. Furthermore, mTOR modulates gene transcription, as well as protein synthesis, thus influencing the differentiation and proliferation of immune cells [120]. Moreover, it also has a significant impact on tumor metabolism [121].

This protein is a 289-kDa serine-threonine kinase consisting of two multiprotein complexes, known as mTOR complex 1 (mTORC1) and mTOR complex 2 (mTORC2) [122,123,124]. Due to the different structural characteristics of mTORC1 and mTORC2, cells respond differently to a range of upstream signals and they are activated by different downstream effectors, producing distinct cellular responses [125].

mTORC1 is comprised of the catalytic subunit (mTOR), the Raptor (regulatory-associated protein of mTOR), mLST8 or GβL (mammalian lethal with Sec13 protein 8), PRAS40 (proline-rich AKT substrate 40 kDa), and Deptor (DEP-domain-containing mTOR-interacting protein) [126]. By promoting several anabolic processes, such as the biosynthesis of proteins, lipids, and organelles, and by limiting autophagy, mTORC1 positively regulates cell growth and proliferation.

mTORC2 consists of mTOR, Rictor (mAVO3) (rapamycin-insensitive companion of mTOR), mSIN1 (mammalian stress-activated protein kinase interacting protein), and mLST8 [126,127,128]. Additionally, several less-conserved proteins, including PRR5/Protor, PRR5L, and DEPTOR (the inhibitory protein DEP domain-containing mTOR-interacting protein) have been discovered to interact with mTORC2 [129]. Biological processes such as cell survival, metabolism, proliferation, and cytoskeleton organization involve mTORC2 [126].

### 2.2. Activation of PI3K/AKT/mTOR in Renal Cancer

One of the most common tumor-related signaling pathways, the PI3K/AKT/mTOR signaling pathway, exhibits aberrant hyperactivation in a range of tumor forms, including breast cancer, colorectal cancer, and RCC, making it a key target for cancer therapy [130].

The molecular oncogenic hallmark of ccRCC is the inactivation of the *von Hippel-Lindau* tumor suppressor gene, which is followed by the subsequent activation of the hypoxia-inducible factors (HIFs) [131]. Up to 95% of sporadic ccRCC have been shown to have biallelic somatic inactivation of *VHL*. The *VHL* gene produces the VHL protein (pVHL), which is a part of the complex involved in the ubiquitination and degradation of HIFs [132], and transcription factors with various roles in the cells are fundamental for the adaptation of cells to oxygen deficiency [133]. The inactivation of *VHL* is carried out through the loss of the 3p chromosome, promoter methylation or mutations [132].

Deregulation of cyclin D1, a cyclin-dependent kinase cofactor necessary for cell cycle progression, is another effect of *VHL* function loss in RCC [134]. mTORC1 regulates the translation of cyclin D by phosphorylating two downstream effectors, p70S6kinase (S6K) and the binding protein for eukaryotic initiation factor 4E (4E-BP1) [135]. Furthermore, mTORC1 and mTORC2 regulate the translation of HIF-1α and HIF-2α subunits, respectively. Moreover, research shows that in ccRCCs with substandard VHL activity, both HIF-1 α and HIF-2α subunits are overexpressed, leading to MAPK (mitogen-activated protein kinase) signaling pathway and AKT/mTOR activation [136].

However, in the different types of RCC, other genetic and/or epigenetic events occur, one of them being the overexpression of VEGF (vascular endothelial growth factor) [18]. MAPK (mitogen-activated protein kinase) and PI3K/AKT can be activated directly by VEGF, which interacts with VEGF-R1 and VEGF-R2, tyrosine receptors found in endothelial cells [137].

Apart from VEGF, other growth factors, such as IGF (insulin-like growth factor), PDGF (platelet-derived growth factor), and EGF (epidermal growth factor) bind to the N-terminal extracellular region of their corresponding transmembrane receptor tyrosine kinases (RTKs), causing the RTKs’ cytoplasmic regions and linking molecules to autophosphorylate tyrosine residues [138]. VEGF-R2 and other growth factor receptors, including EGFR and IGFR, in ccRCC cells suffer dimerization and activation due to the overexpression of VEGF, EGF, and IGF. When activated, these tyrosine kinase receptors will activate either the PI3K/AKT/mTOR signaling pathway or the RAS/mitogen-activated protein kinase /extracellular signal-regulated kinase (RAS/MEK/ERK) pathway to increase the production of HIFs, thereby accelerating the growth of the tumor [139]. After its allosteric activation, PI3K is recruited to the RTKs via contacts between the p85 SH2 domains and phospho-Tyr residues on members of the RTK complex [138].

Through interactions of their pleckstrin homology domains with PtdIns(3,4,5)P3 produced by PI3K, AKT and its upstream kinase, PDK1 (3-phosphoinositide-dependent protein kinase-1), are recruited to the inner cell membrane. Subsequently, PDK1 phosphorylates AKT at Thr308. AKT is most effectively activated when mTORC2 and other kinases phosphorylate Ser473 in the regulation hydrophobic region of AKT [140]. Once phosphorylated, AKT is activated and translocates from the cell membrane to different cell compartments where it phosphorylates a variety of downstream substrates. Cell survival, development, metabolism, tumorigenesis, and metastasis are just a few of the many physiologic and pathologic cellular processes that are regulated by activated AKT’s phosphorylation of several substrates [90].

The activation of mTORC1 mediated by AKT promotes protein translation and lipid or nucleotide synthesis. Moreover, AKT phosphorylates and inhibits the GTPase-activating protein for the RHEB (Ras-related small G protein RHEB) and TSC1/TSC2 (tuberous sclerosis complex 1/2) [141].

Moreover, upon activation, activated mTORC1 inhibits apoptosis through its downstream effector proteins such as p70S6K1, allowing the cell cycle to go into the G1 phase [137].

In ccRCC, mutations were also found in several mTOR inhibitors, including PTEN (phosphatase and tensin homolog deleted from chromosome 10) and TSC1/2, highlighting the crucial impact of the mTOR signaling the development of ccRCC. Located on chromosome 10q23.3, tumor suppressor gene *PTEN* participates in cellular signaling pathways that regulate and control DNA repair, cell growth, proliferation, senescence, and apoptosis, as well as genic mutation [142]. Research related to PTEN, an inhibitor of PI3K is epigenetically reduced in ccRCC, reveals that elevated miR-21 is associated with reduced *PTEN* expression, favoring cell proliferation and migration [143].

Additionally, a study by Que W et al. showed that reduced *PTEN* expression is a factor that negatively affects the overall survival of kidney cancer patients. Moreover, a positive correlation was found between the decrease in *PTEN* expression and the development and severity of renal cancer [142]. On the other hand, Hager M et al. revealed that in the initial stage of renal cell carcinoma, *PTEN* was underexpressed, but the expression pattern of *PTEN* was not predictive of the prognosis for patient survival [144].

Mutations in either *TSC1* (encodes hamartin) or *TSC2* (encodes tuberin) determine loss of the suppressor functions of these genes that are located on chromosomes 9q34 and 16p13, respectively, will activate mTOR, promoting aberrant cellular growth, proliferation, and protein synthesis [145,146]. Apart from activating mTOR, *TSC1/TSC2* inactivating mutations also result in increased HIFs translation [147].

Furthermore, current research showed that RCC with leiomyomatous stroma (RCCLS) exhibit frequent *TSC1* or *TSC2* mutations, and it is thought that ESC (eosinophilic solid cystic)-RCC may be pathognomonic for the mutations of these genes [148].

## 3. Reprogramming Glucose, Lipid, and Amino Acids Metabolism in Renal Cancer

### 3.1. Glucose Metabolism in Renal Cancer

RCC is a malignancy of dysregulated metabolism, while mTOR is an important regulator of cell metabolism [1]. It is well known that most patients with cancer have changes in nutrition status [149]. Therefore, the daily consumption of animal fats and red meat is correlated with increased cancer mortality, because these types of food induce the synthesis of IGF1 (insulin growth factor 1), an activator of the mTOR pathway which is associated with tumor progression [150].

Insulin can activate the PI3K signaling pathway, which controls cell metabolism and plays an important role in the proliferation and survival of the cell, ultimately deciding its fate [151]. The insulin-dependent control of systemic and cellular metabolism depends on the PI3K/AKT pathway, with AKT2 representing the primary isoform needed for insulin roles in metabolisms. Serine residues on insulin receptor substrates may be phosphorylated by mTORC1, resulting in their ubiquitylation and proteolytic degradation. Chronically increased levels of insulin may promote the activation of the insulin receptor on preneoplastic cells directly or indirectly by promoting the synthesis of IGF1, with studies performed on xenograft models showing that insulin and IGF1 promote cell proliferation and decrease apoptosis, therefore leading to tumor growth [152].

Moreover, mTORC1 stimulates insulin secretion and fatty acids (FA) oxidation in muscles, while in the liver it stimulates gluconeogenesis and reduces the formation of ketone bodies [153].

The most important isoform of PI3K regarding its role in insulin signaling is PI3Kα, playing an important role in insulin resistance. Meanwhile, inflammation, fatty liver, insulin resistance, and diet-related obesity have been linked to PI3Kγ [154].

Through the nuclear receptor PPAR and signaling via ribosomal protein S6 kinase beta-1 (S6K1 or p70S6 kinase) mTOR regulates adipogenesis, reducing lipolysis and promoting adipocyte clustering, leading to accumulation. mTORC1 regulates the activity of SREBP-1c (sterol regulatory element-binding protein-1c), a transcription factor that stimulates the synthesis of FA and cholesterol. Moreover, mTORC1 stimulates the synthesis of triglycerides by modulating the phosphorylation of a phosphatase lipin 1 (Lpin1) [153].

Metabolic reprogramming plays a pivotal role in cancer progression because it allows the cells to survive in nutrient- and oxygen-deprived conditions, become tolerant to stress, and have the capacity to metastasize in different body sites [155,156].

Moreover, in ccRCC metabolic reprogramming of glucose, fatty acid metabolism and tricarboxylic acid cycle have been identified, with glycolysis being the most frequent metabolic alteration in RCC [157,158]. In renal cancer, reprogramming metabolism takes place in stromal cells [155].

Normally, most mammalian tissues, including the renal cortex with proximal and distal tubules, have an O_2_ content of 2–9%. Additionally, in normal conditions, our kidneys can metabolize free fatty acids (FFAs), glycerol, lactate, pyruvate, 3-hydroxybutyrate, glutamine, amino acids (proline, glutamine), and Krebs cycle intermediates (citrate, α-ketoglutarate). In the proximal tubule reabsorption of the mentioned compounds occurs in a percentage of 70%. Moreover, FFAs-β-oxidation occurs in the mitochondria-proximal tubule cells [159]. For example, excessive kidney FA loads induce proximal tubular epithelial cells toxicity and lead to kidney disease development such as tissue fibrosis [160].

In contrast, the kidney medulla is physiologically less oxygenated, with 1.4–2.8% of O_2_ [161]. In kidneys, oxygen is used differently, from the cortex to the medulla. Therefore, a difference in metabolism from the cortical to medullary nephron segments exists. In addition, proximal tubules have a highly aerobic metabolism, where oxidative phosphorylation is used to generate ATP, while the distal nephron has decreased aerobic conditions [162]. In healthy subjects, in kidney tubules, ATP is obtained mostly by fatty acid β-oxidation (FAO) [163].

RCC is characterized by several metabolic dysregulations including oxygen sensing (VHL/HIF pathway), glucose transporters (GLUT1 and GLUT4) energy sensing, and energy nutrient sensing cascade [153]. RCC presents cellular alterations in cellular sugars, lipids, amino acids, and nucleic acids’ metabolism [157,164]. Therefore, dysregulation of cellular metabolism is an important cancer hallmark that contributes to tumor initiation, progression, and tumor heterogeneity [165].

ccRCC is characterized by the loss of the *VHL* gene and further overexpression of hypoxia-inducible factor 1 alpha (HIF-1α), involved in cancer cell metabolic reprogramming [166]. On the other hand, in the pathogenesis of ccp-RCC, HIF is also activated by non-VHL-dependent mechanisms because immunohistochemical results revealed the presence of HIF-1 and GLUT-1 [167].

Tumors can reprogram metabolic pathways involved in nutrient uptake via a phenomenon called reprogramming. These molecular changes are possible because tumor suppressor genes are lost, and oncogenes are activated. Because multiple metabolic pathways suffer alterations in RCC, this malignant disease is considered a “metabolic disease” [168].

Therefore, ccRCC- metabolic dysregulations involve aerobic glycolysis, Krebs cycle, pentose phosphate pathway, fatty acid synthesis and beta-oxidation, oxidative phosphorylation, glutathione, and glutamine metabolism [169,170,171]. The genes involved in glycolysis, the pentose phosphate pathway, glutamine metabolism, and lipogenesis are upregulated [172]. In cancer cells, the tumor suppressor of HIF, pVHL, will bind and degrade HIF. Moreover, in ccRCC, *VHL*/pVHL is mutated and its functions are lost, leading to HIF protein overexpression [173]. HIF was also linked to renal medullary carcinoma [174].

In ccRCC, HIF will induce the transcription of genes necessary for glycolysis and lactate metabolism [175]. HIF has two subunits, alpha, and beta. The family of enzymes, prolyl-4-hydroxylases (PHD1–3), catalyze the hydroxylation of proline residues from HIF-alpha subunits, activating hundreds of genes involved in angiogenesis, proliferation, survival, metabolism, apoptosis [176] motility, cytoskeletal structure, cell adhesion, and cellular metabolism [177]. HIFs are O_2_-sensitive transcription factors that adapt RCC cells to hypoxia, regarding VEGF production, metabolic reprogramming of cellular glucose, and also energy metabolism [178]). In addition, via transcriptional regulation, HIF acts on VEGF, PDFG, EGF, GLUT 1, TGF-alpha, and erythropoietin [179].

Therefore, HIF-1α contributes to cancer cell proliferation, migration, and survival (de Carvalho PA, 2021) and other processes that include GLUT 1 and cyclin D [166]. In normal tubular cells, HIF-1α is present, while HIF-2α is found in pVHL-detective tubular renal cells [136].

HIF-1α protein was detected in VHL-deficient renal epithelial cells, and also ccRCC, suggesting its implications in cancer progression [180]. Therefore, mutations in *VHL* and alterations in its downstream pathways play crucial roles in RCC development and progression [181]. In addition, hypoxia induces angiogenesis, which plays a significant role in RCC progression [182].

Moreover, HIF activates Ras, leading to Krebs cycle substrate accumulation, including fumarate and also PI3K/AKT/mTOR activation [183]. In RCC, HIF-1α controls cancer cell metabolism, by increasing glucose uptake, through glycolytic and pentose phosphate pathways. In addition, Lucarelli G and his research team detected in tumor tissue increased levels of glucose, and intermediates of upstream glycolytic compounds such as glucose-6-phosphate and fructose-6-phosphate. Instead, the levels of 3-phosphoglycerate, 2-phosphoglycerate, and phosphoenolpyruvate, downstream intermediates were decreased [184].

RAS promotes glycolysis in renal cancer cells because increases the expression of GLUT 1, which further conduces to lactate production by the Warburg effect [185]. Moreover, hyperinsulinemia ensures the energy for tumor cells and sustains their growth [186]. The Warburg effect requires increased glucose levels. For this reason, tumor cells upregulate the expression of glucose transporters (GLUTs) sodium-dependent glucose transporters (SGLTs) [187].

Furthermore, HIFs induce the expression of several proteins and enzymes in glucose metabolism such as GLUT1, LDH, PDK1 (pyruvate dehydrogenase kinase), HK (Hexokinase), and PGK (phosphoglycerate kinase). HIFs also mediate the inhibition of the Krebs cycle and further oxidative phosphorylation [168]. In ccRCC, pVHL invalidation stabilizes HIF in the presence of oxygen, stimulating further glycolytic gene expression [188].

It is considered that *VHL* inactivation induces in mice and human renal cysts formation but is not enough to cause ccRCC [189].

In most cancer cells and especially in high-grade ccRCC tissues, the Warburg effect is overexpressed [190]. In the presence of oxygen, cancer cells consume glucose and produce lactate. Studies performed in vivo using carbon labeling revealed that transformed cells and T cells use glucose in an anaerobic mode [191]. RCC is correlated with increased production of lactate and nitric oxide (NO) [192]. An elevated level of the enzyme LDHA, implicated in the reduction of pyruvate to lactate was detected in renal cancer cells compared with normal cultures [193].

Glucose-6-phosphate (G6P), an important enzyme used to produce energy as ATP and NADH, is used for glycogenesis and by the pentose phosphate pathway (PPP). The activity of this enzyme is elevated in cells undergoing normal or neoplastic cell growth, including RCC [194]. Mitochondrial metabolism is involved in cancer cell growth, providing precursors to synthesize macromolecules. Therefore, citrate and acetyl-CoA obtained from glucose metabolism are used in tumorigenesis for lipid synthesis [195]. The glycolytic intermediates are used by cancer cells to synthesize lipids, amino acids, and nucleic acids [196]. The isoform pyruvate kinase M2 (PKM2) activity is essential for cancer survival, including RCC, which has been overexpressed in the proximal renal tubule [197].

Cancer cell metabolism is associated with the dysregulation of GLUT1, whose mRNA levels are influenced by microRNAs. In this context, Morais M. and his research team evaluated the levels of intra and extracellular levels of microRNAs and protein levels in three ccRCC levels. The study reported decreased intracellular levels of microRNAs and increased levels of GLUT 1. The study also detected an increased amount of glucose consumption correlated with higher levels of lactate production [198].

AKT plays a key role, in both glycolytic and oxidative metabolism, because its activation will increase ATP production, while its deprivation reduces ATP levels. Moreover, AKT enhances the expression of glucose transporters, increasing the coupling between oxidative phosphorylation and glycolysis, leading to HIF1α and HK2 accumulation, and also activation of phosphofructokinase-2 (PFK-2) [199].

Fumarate hydratase (FH), an enzyme from the Krebs cycle catalyzes the conversion of fumarate to malate. Mutations of this enzyme will lead to malate accumulation, correlated with important metabolic changes [200]. A study by Xin Ge et al. revealed that in patients diagnosed with type 2 papillary renal cell carcinoma, FH deficiency leads to fumarate accumulation, which will inhibit the negative regulator PTEN, therefore promoting tumorigenesis and therapeutic resistance [201]. Moreover, Wang Q and his research team also detected in papillary renal cell carcinoma that the most up-regulated gene was lactate dehydrogenase A (LDHA), while FH is downregulated, both involved in ATP generation [202]. Additionally, hereditary leiomyomatosis and renal cell carcinoma are characterized by germline mutation of the Krebs cycle enzyme, fumarate hydratase [203].

Furthermore, Sudarshan S. and co-workers reported that FH depletion is correlated with acute upregulation of GLUT1 protein, glucose uptake, and further lactic acid production. In addition, succinate accumulation may have similar effects. Taking into consideration these aspects, cancer renal cells will use glycolysis as a source of ATP when TCA enzyme expression is reduced [204]. Moreover, in RCC FH deficiency seems to be correlated with increased levels of HIF-1α and HIF-2α [205].

Increased production of lactic acid is correlated with cancer metastasis, via TGF-β2 pathway. Moreover, lactic acid stimulates endothelial cells to produce VEGF and can generate pyruvate that is converted by decarboxylation into acetyl-CoA, which will be further used by cancer cells for cholesterol and FA synthesis [206].

The pentose phosphate pathway (PPP) is a metabolic pathway where glucose is used not to obtain ATP; instead, it generates ribose 5-phosphate and NADPH. NADPH is necessary for GSH regeneration from GSSG. In ccRCC, a higher GSH/GSSG ratio was detected, important for GSH conversion. Additionally, glucose-6-dehydrogenase, the first enzyme from the pentose pathway is elevated in ccRCC patients [207].

PPP occurs in ccRCC cells to obtain NAPDH and ribose-5-phosphate, used further for nucleotide and lipid biosynthesis. Moreover, NAPDH produced by renal cancer cells is used to fight against reactive oxygen species (ROS) [208].

Increased glycolysis leads to ATP production used for renal cancer cell proliferation. Renal cancer cell-FH deficiency conduces to decreased levels of p53 and increased production of FA biosynthesis because the activity of acetyl-CoA carboxylase is decreased [209].

Furthermore, ccRCC is characterized by increased glycogen and lipid content [210]. It is already known that glycogen represents the mammalian storage of alpha-glucose, bounded 1–4 and 1–6. The liver, kidney, muscle, and brain are the most important energy-consuming organs in normal human physiological processes. In pathological conditions such as cancer, increased glycogen benefits tumor microenvironment development [211]. Schaeffeler E. and co-workers isolated cells from ccRCC and observed that primary cultures are characterized by lipid and glycogen storages and also aerobic glycolysis/lactate fermentation [193].

Glycogen phosphorylase (GP), glycogen synthase (GYS), phosphoglucomutase 1 (PGM1), and protein phosphatase 1 regulatory 3 (PPP1R3) are enzymes involved in glycogen degradation and synthesis. In cancer cells, hypoxia, tumor suppressors, and oncogenes regulate glycogen metabolism, favoring its degradation [212].

Responsible for dysregulation in glycogen metabolism are histone modification [212]. Glycogen synthase kinase-3 (GSK-3) is a key serine/threonine kinase involved in glycogen metabolism that regulates the cell cycle and proliferation. GSK-3 dysregulation leads to its involvement in the pathogenesis of many human diseases such as type 2 diabetes, bipolar disorder, inflammation, and cancer [213]. In hypoxic conditions, oxidative phosphorylation switches to anaerobic metabolism [214].

Taking this into consideration, glycolysis intermediates represent the precursors for lipids, amino acids, and nucleotide synthesis necessary for tumor cell survival and proliferation [215].

### 3.2. Lipid Metabolism in Renal Cancer

Alteration of the lipid metabolism allows tumor cells to survive [216]. In normal renal proximal tubule cells, FA via beta-oxidation ensures the ATP necessary for renal sodium reabsorption [217]. Fatty acids and cholesterol synthesis were overexpressed in ccRCC and correlated with tumor aggressiveness and poor prognosis [218]. Additionally, lipid droplet formation is a defining histological feature in ccRCC.

Cancer lipid metabolic reprogramming is implicated in the biosynthesis of fatty acids, ceramides, and sphingolipids, which regulates various signaling pathways important for cancer cells. Therefore, cancer cells have increased de novo synthesis and β-oxidation of FFAs [219]. Through HIF-dependent modulation of proteins involved in fatty acids metabolism (uptake, synthesis, usage, storage), in hypoxic conditions, lipogenesis is enhanced. HIF-1 activates the gene that encodes the synthesis of PPARγ (peroxisome proliferator-activated receptor gamma) transcription factor, which stimulates the uptake of FA and the synthesis of TAGs [220]. Furthermore, in cancer cells, HIF-1 directly targets AGPAT2 (acylglycerol-3-phosphate acyltransferase 2), which enhances the TAG biosynthesis pathway, resulting in increased storage of lipids [221].

Młynarczyk G and his research team detected in ccRCC tissues accumulation of sphingosine, sphingosine-1-phosphate (S1P), ceramide, dihydrosphingosine, and dihydroceramide compared with healthy subjects [222]. In ccRCC, lipid accumulation will affect cellular energy homeostasis and lipid signal transduction [219], contributing to cancer cell proliferation, progression, and metastasis [223,224].

In cancer cells, lipolysis is increased leading to an increased amount of FA accumulation and further LDL formation. In renal hypoxic conditions, LDL is accumulated [225].

Li W. and his research team using 10 pairs of cancerous and adjacent normal tissues collected from ccRCC patients detected 28 lipid classes. The study reported that the most abundant lipids with increased levels were triacylglycerol, diacylglycerol, phosphatidylcholine, and phosphatidylethanolamine. Moreover, among them, esterified cholesteryl presented a considerably increased level in tumoral cells compared with normal samples. Activated saturated and unsaturated FA bound by carnitine also had increased levels in tumoral renal cells versus healthy cells [226].

Moreover, in ccRCC increased levels of cholesterol disrupt the lipid metabolism in T cells, leading to the suppression of immune function, and protecting the cells against various treatments [227]. Cholesterol production of cholesterol and synthesis of fatty acids was also found elevated in tumors with WT1 mutations [228].

Dysregulation of the following enzymes—hydroxy acyl-CoA dehydrogenase α-subunit (HADHA), acetyl-CoA acetyltransferase 1 (ACAT1), ATP-citrate lyase (ACLY), and ATP synthase β- subunit (ATP5B)—were also detected in RCC (Liu S/2019). HADHA represents the alpha subunit of the mitochondrial trifunctional protein that catalyzes the last three stages of long chain FFAs-β-oxidation [229].

Additionally, HIFs influence the lipid metabolism of renal cancer cells, acting on the enzyme implicated in the mitochondrial fatty acid transport, carnitine pal-mitoyltransferase 1A (CPT1A), which is the rate-limiting enzyme. Moreover, HIF-1 and HIF-2 repress CPT1A, forcing FA to form droplets for storage [230].

### 3.3. Amino Acids Metabolism in Renal Cancer

Amino acids are used not only for protein biosynthesis but are also important intermediates for carbon and nitrogen biosynthesis, ATP generation, S-adenosyl methionine, and antioxidant production [231]. Yang H. and colleagues demonstrated that element-binding protein 1 (SREBP1) is overexpressed in ccRCC cell lines, and is important for ccRCC lipid desaturation and cell growth. In addition, SREBP1 activates the NF-kB pathway [232].

The “kidney-type” glutaminase (GLS1) is an enzyme that plays a key role in glutaminolysis [233]. RCC cells utilize glucose and glutamine to support cell growth and proliferation [234].

Glutamine is essential for many cancer cells’ fundamental functions such as the production of antioxidants, maintaining mitochondrial metabolism, and activation of cell signaling. In mitochondria, the dehydrogenases of glutamate to α-ketoglutarate are catalyzed by glutamate dehydrogenase, regulated by ADP-ribosylation, a process mediated by mitochondria protein sirtuin 4 (SIRT4) [235].

**Figure 1:**
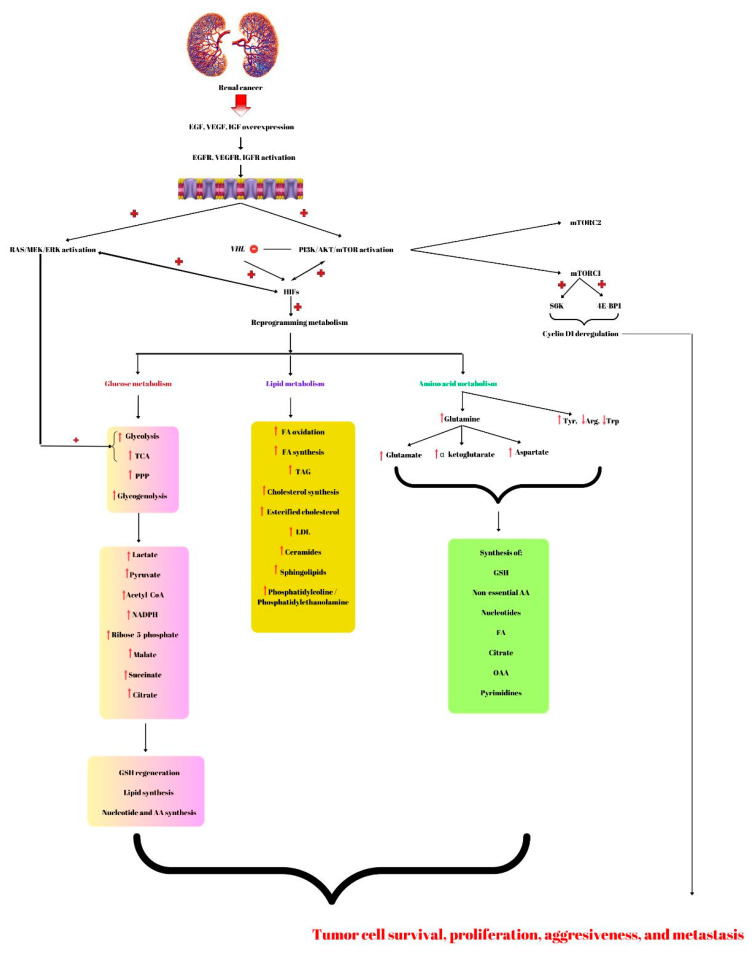
Renal cancer is characterized by overexpression of growth factors; epidermal growth factor (EGF), vascular endothelial growth factor (VEGF), and insulin growth factor (IGF) that will induce the activation of the phosphatidyl-3-kinase-protein-kinase B-mammalian target of rapamycin (PI3K/AKT/mTOR) and RAS/MEK/ERK (RAS/mitogen-activated protein kinase/extracellular signal-regulated kinase). Further, this molecular signaling pathway inhibits the Von Hippel-Lindau (VHL) gene leading to the activation of hypoxia-inducible factors (HIFs) involved in renal cancer-reprogramming metabolism. Therefore, regarding glucose metabolism, this malignancy has increased glycolysis, glycogenolysis, pentose phosphate pathway (PPP), and increased tricarboxylic acid cycle (TCA), leading to lactate, pyruvate, NADPH, ribose-5-phosphate, malate, succinate, citrate, and acetyl-CoA accumulation. Additionally, the RAS/MEK/ERK signaling pathway promotes glycolysis and TCA. The compounds that resulted from altering glucose metabolism are involved in glutathione (GSH) regeneration, lipids, nucleotides, and amino acids (AA) biosynthesis. Fatty acids (FA) are used to produce energy by β-oxidation. Lipid metabolism alterations also include synthesis of FA, TAG (triacylglycerols), cholesterol, ceramides, sphingolipids, and phosphatidylcoline/phosphatidylethanolamine. Amino acids such as glutamine can suffer several modifications forming glutamate, α-ketoglutarate, and aspartate. The intermediates together with increased Tyr (tyrosine) and decreased Arg (arginine) and Trp (tryptophan) will be used by renal cancer cells for the synthesis of new AA, nucleotides, FA, citrate, oxaloacetate (OAA), and pyrimidine bases. All these compounds that result from the altered pathway lead to cancer cell survival, aggressiveness, and metastasis. These characteristics of malignant tumors are also promoted by cyclin D1 deregulation produced by mTORC1 by activating S6K and 4E-BP1. + represents activation; − means inhibited.

Glutamine is one of the most abundant plasma amino acids and an important compound used to produce energy in ccRCC. Glutamine can enter the cell via glutamine transporter (ASCT2) where it can suffer transamination with α-ketoglutarate and NADH formation. Moreover, glutamine can be used for NADPH, ammonium, and other non-essential amino acid production. Further, these compounds derive from glutamine and are used for amino acids, nucleotides, FA, citrate, and oxaloacetate synthesis (Figure 1) [236]. Moreover, glutamine is a donor of nitrogen necessary for nucleotides, hexosamines, and non-essential amino acid biosynthesis [237].

From glutamine, aspartate can be obtained, as it is involved in pyrimidine biosynthesis and GSH for cellular redox protection. In addition, glutamine can contribute to cancer resistance against therapy. For example, mTORC1 activation induces glutamine anaplerosis by repressing SIRT4 transcription leading to glutamate dehydrogenase activation [238]. In RCCs, HIFs support and stimulate the reductive glutamine metabolism [239,240]. By inducing the expression of GLS1 (glutaminase 1), HIF-1 augments the level of α-ketoglutarate in cancers, allowing increased citrate synthesis and increased FA/lipid production [241]. Therefore, by acting on AA metabolism, HIFs influence glutamine signaling and enhance tumor progression [242].

Wang J and his research team illustrated that two enzymes involved in tyrosine metabolism, homogentisate 1,2-dioxygenase (HGD), and glutathione S-transferase zeta 1 (GSTZ1) were downregulated in three renal cancer types (KIRC, KIRP, and KICH) of tissues versus healthy ones. In KIRC, these enzymes promote aerobic glycolysis, coordinate amino acid and energy metabolism, and activate the tumor cell cycle, leading to cancer progression [243].

While in healthy status, arginine is a semi-essential or conditionally essential amino acid necessary for development [244,245]. In RCC, amino acids arginine and tryptophan have decreased levels (Yuan Y/2022). Moreover, the mTORC1/4E-BP axis regulates the synthesis of aspartate, asparagine, and serine by modulating mRNA translation [246].

Glutathione, the cell-atypical non-enzymatic tripeptide protects our cells against hydroperoxides, hydrogen peroxide, and lipids peroxides. Miess H. and co-workers reported in the MYC-dependent mouse model of renal cancer that GSH activation blocks tumor growth [247].

The enzyme carbonic anhydrase IX (CA9) is a pH-regulating transmembrane protein, over-expressed in solid tumors, including ccRCC. Xu J and his colleagues confirmed that ccRCC-CA9 mRNA expression was significantly elevated. It was observed that CA9 knockdown upregulates proteins involved in oxidative phosphorylation, and increases mitochondrial biogenesis, leading to the reversal of the Warburg phenotype and in the end cancer cell growth inhibition. The study conducted by Xu J observed that CA9 knockdown upregulated the enzyme mitochondrial arginase 2 (ARG2), resulting in putrescine accumulation. The obtained diamine will further suppress ccRCC proliferation [44].

In WT, numerous other metabolic proteins, such as aldehyde dehydrogenases and proteins involved in propanoate and butanoate metabolism, long-chain fatty acid metabolism, and the breakdown of the branched-chain amino acids valine, leucine, and isoleucine, were also found to be reduced versus normal tissue. It is unclear, though, if these modifications also exist in primary, untreated WT since the tissue samples analyzed by Hammer E et al. were taken after chemotherapy treatment [248].

The metabolic reprogramming in chRCC has been discovered using proteome profiling and includes halted gluconeogenesis, downregulated oxidative phosphorylation, and altered fatty acid and amino acid metabolism. In chRCC, an analogous anticorrelation between transcripts and proteins (as in pRCC) was discovered. Compared to ccRCC and pRCC, chRCC exhibits a much-reduced microvessel density and a slower rate of glucose uptake, suggesting that chRCC cells favor a different mode of nutrient uptake to make up for the microenvironment’s lack of nutrients. In order to obtain extracellular macromolecules as a source of nutrition for cell survival and proliferation, chRCC cells can activate the endocytosis and downstream lysosomal pathways, which is demonstrated by the abundance increase in proteins implicated in these pathways and their enzymatic activity [207].

A limitation of the present review is that most of the discussed metabolic reprogramming refers to RCC subtypes because of the limited number of papers in the literature about the other types of renal cancer, taking into consideration that RCC represents the most common malignancy of the kidney. However, having a better understanding of the molecular events and signaling pathways involved in renal cancer could help improve the management and the tools used to diagnose, treat, and monitor renal cancer patients. The management of renal cancer is difficult in all aspects, from diagnosis to therapy and follow-up of the patients. Usually, the differential diagnosis of benign or malignant tumors is based on imaging and a biopsy. Nevertheless, these methods are not always efficient in renal cancer because of the heterogeneity of the tumors. Therefore, additional diagnosis and monitoring tools, such as artificial intelligence, genomics, and radiogenomics have been developed in recent years, which together with the greater knowledge of the molecular events associated with the histopathology aspects, could provide new perspectives into clinical practice [249,250].

## 4. Conclusions

Although the survival rate of renal cancer improved in the last decades, the incidence of this malignancy has increased, with renal cell carcinoma being the most frequent type of renal cancer in adult patients. RCC is considered to be a malignancy of dysregulated metabolism. Meanwhile, PI3K/AKT/mTOR signaling pathway control metabolism and a wide range of cellular processes, including survival, proliferation, growth, and angiogenesis. Moreover, the molecular oncogenic hallmark of ccRCC is the inactivation of the *VHL* tumor suppressor gene. Subsequently, HIFs, crucial transcription factors are activated, and their translation is regulated by mTORC1 and mTORC2. Consequently, glycolysis, glycogenolysis, and pentose phosphate pathway are increased, fatty acids oxidation is promoted, and glutamine metabolism is overstimulated, leading to products that will sustain tumor cell development. In conclusion, renal cell carcinoma is influenced by several signaling pathways, including PI3K/AKT/mTOR and the VHL/HIF axis. Hence, the inhibition of the key members of this signaling pathway could offer new therapy perspectives.

Furthermore, the integration of renal cancer histology, genetic features of each type of malignancy, omics, and molecular signaling can improve the understanding of the basic biological mechanisms underlying tumors, which are still greatly unknown and which could provide new instruments for the detection and treatment of renal cancer.

## Figures and Tables

**Table 1:** IACR/IARC/WHO classification of renal tumors.

Renal Cell Tumors	
**Clear Cell Renal Tumors**	
8310/3	Clear cell renal cell carcinoma
8316/1	Multilocular cystic renal neoplasm of low malignant potential
Papillary renal tumors	
8260/0	Papillary adenoma
8260/3	Papillary renal cell carcinoma a
Oncocytic and chromophobe renal tumors	
8290/0	Oncocytoma
8317/3	Chromophobe cell renal carcinoma
	Other oncocytic tumors of the kidney
Collecting duct tumors	
8319/3	Collecting duct carcinoma
Other renal tumors	
8323/1	Clear cell papillary renal cell tumors
8480/3	Mucinous tubular and spindle cell carcinoma
8316/3	Tubulocystic renal cell carcinoma
8316/3	Acquired cystic disease–associated renal cell carcinoma
8311/3	Eosinophilic solid and cystic renal cell carcinoma
8312/3	Renal cell carcinoma, NOS
Molecularly defined renal carcinomas	
8311/3	*TFE3*-rearranged renal cell carcinomas
8311/3	*TFEB*-altered renal cell carcinomas
8311/3	*ELOC* (formerly *TCEB1*)-mutated renal cell carcinoma
8311/3	Fumarate hydratase–deficient renal cell carcinoma
8311/3	Hereditary leiomyomatosis and renal cell carcinoma syndrome–associated renal cell carcinoma
8311/3	Succinate dehydrogenase–deficient renal cell carcinoma
8311/3	*ALK*-rearranged renal cell carcinomas
8510/3	Medullary carcinoma, NOS
8510/3	SMARCB1-deficient medullary-like renal cell carcinoma
8510/3	SMARCB1-deficient undifferentiated renal cell carcinoma, NOS
8510/3	SMARCB1-deficient dedifferentiated renal cell carcinomas of other specific subtypes
Metanephric tumors	
8325/0	Metanephric adenoma
9013/0	Metanephric adenofibroma
8935/1	Metanephric stromal tumor
Mixed epithelial and stromal renal tumors	
8959/0	Mixed epithelial and stromal tumor
8959/0	Adult cystic nephroma
8959/0	Pediatric cystic nephroma
Renal mesenchymal tumors	
Adult renal mesenchymal tumors	
8860/0	Angiomyolipoma
8860/0	Oncocytic angiomyolipoma
8860/0	Angiomyolipoma with epithelial cysts
8860/1	Angiomyolipoma, epithelioid
9161/1	Hemangioblastoma
8361/0	Juxtaglomerular tumor
8361/0	Functioning juxtaglomerular cell tumor
8361/0	Nonfunctioning juxtaglomerular cell tumor
8966/0	Renomedullary interstitial cell tumor
Pediatric renal mesenchymal tumors	
8967/0	Ossifying renal tumor of infancy
8960/1	Mesoblastic nephroma
8960/1	Classic congenital mesoblastic nephroma
8960/1	Cellular congenital mesoblastic nephroma
8960/1	Mixed congenital mesoblastic nephroma
8963/3	Malignant rhabdoid tumor of the kidney
8964/3	Clear cell sarcoma of the kidney
Embryonal neoplasms of the kidney	
Nephroblastic tumors	
	Nephrogenic rests
	Perilobar nephrogenic rests
	Intralobar nephrogenic rests
	Nephroblastomatosis
8959/1	Cystic partially differentiated nephroblastoma
8960/3	Nephroblastoma
Miscellaneous renal tumors	
Germ cell tumors of the kidney	
9084/0	Prepubertal-type teratoma
9084/3	Teratoma with carcinoid (neuroendocrine tumor)
9071/3	Yolk sac tumor, NOS
9085/3	Mixed teratoma–yolk sac tumor

This combined IACR/IARC/WHO classification of the renal tumors tends to integrate multiple criteria, including the behavior of the tumors [5]: 0—benign tumors; 1—uncertain, unspecified, or borderline behavior; 2—grade III intraepithelial neoplasia and carcinoma in situ; 3—primitive malignant tumors; 6—metastatic malignant tumors.

**Table 2:** Summary of the roles of PI3K classes.

PI3K Class	Involved in/Effects	References
Class I	Second messenger for recruitment of cytoplasmic proteins	[94]
	Survival and maturation of B cell follicles	[95]
	Promote inflammation	[100]
	Modulator of heart contractility	[100]
	Allergies	[100]
Class II	Embryonic development	[104]
	Insulin production and signaling	[104]
	Angiogenesis	[104]
	Primary cilium function	[104]
	Mitotic progression	[96,106]
	Tumor proliferation and invasion	[107]
Class III	Endosome fusion	[114]
	Autophagy and phagosome formation	[114]
	Macrophage phagocytosis	[114]
